# Establishing effective cross-disciplinary collaboration: Combining simple rules for reproducible computational research, a good data management plan, and good research practice

**DOI:** 10.1371/journal.pcbi.1011052

**Published:** 2023-04-27

**Authors:** Bogna Stawarczyk, Małgorzata Roos

**Affiliations:** 1 Department of Prosthetic Dentistry, Ludwig-Maximilians-University Munich, Munich, Germany; 2 Epidemiology, Biostatistics and Prevention Institute, University of Zurich, Zurich, Switzerland; University of Virginia, UNITED STATES

Cross-disciplinary collaborative research projects are often essential in many fields for making scientific progress, but they are notoriously difficult to conceptualize and design. The recent increased focus on reproducible science makes the design of such research projects even more challenging. We demonstrate that simple rules provided by three editorial articles from the series “Ten Simple Rules” greatly impacted the process of writing of our research proposal, leading to a design that can serve researchers from a wide range of disciplines to conceptualize their own collaborative projects that promote reproducible science.

## Ten simple rules

**Fig 1:**
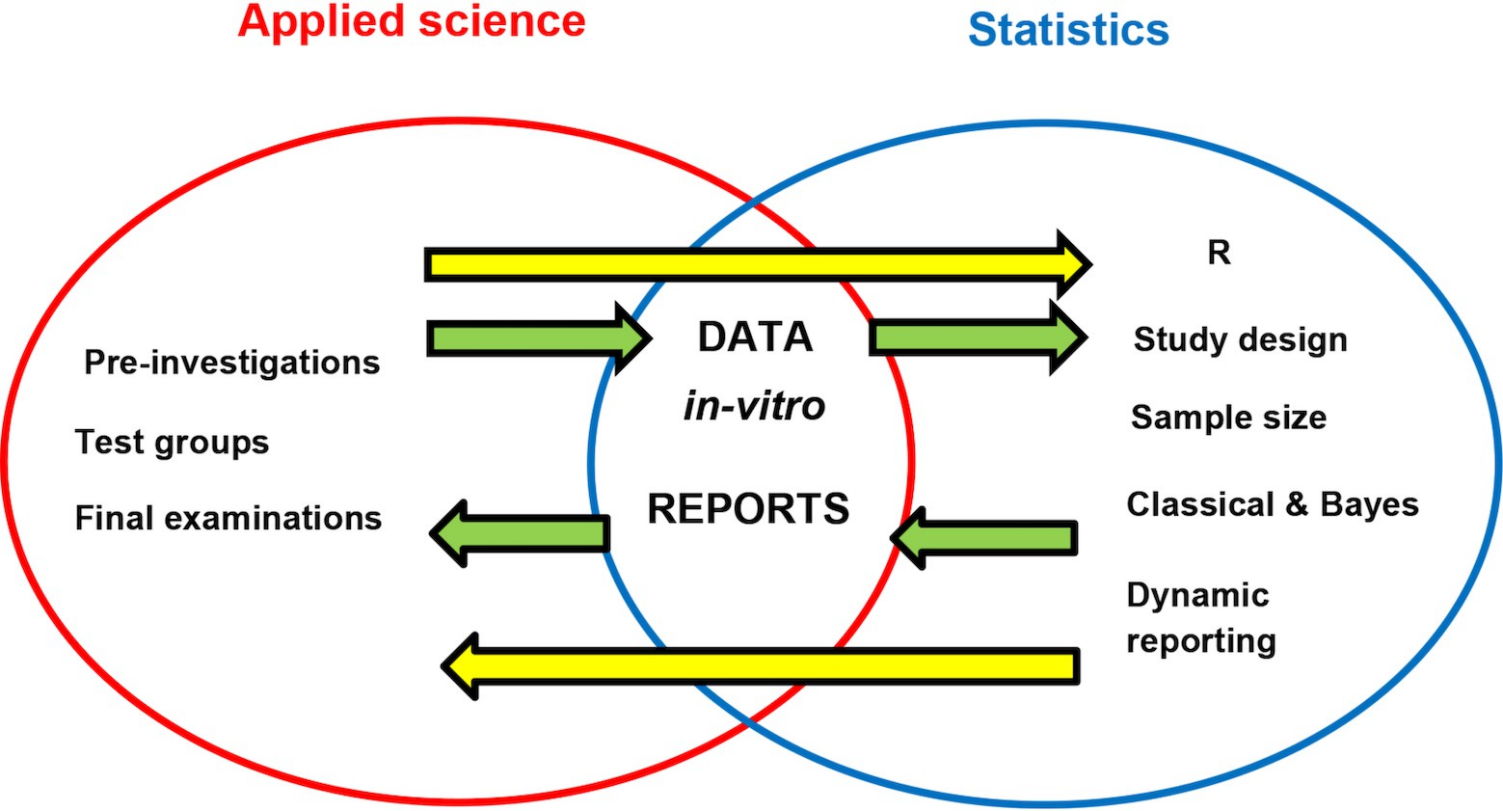
Project with two equal partners from an applied science on the left and statistics on the right. Within this project, both partners must accomplish the tasks listed. Both partners communicate either directly (yellow arrows) or indirectly (green arrows) through provisional results including in vitro raw data and statistical reports.

A decade ago, we conceived an international cross-disciplinary collaborative project focused on reproducible dental materials research with dynamic statistics. Only in summer 2022 did we find favorable circumstances to formalize our project (Fig 1) and write a proposal. We had to cope with considerable time pressure to meet the deadline for the submission of scientific projects and in this we benefited from three editorial articles from the series “Ten Simple Rules”. The “Ten Simple Rules” series of articles is written by experts who share simple practical rules for mastering different professional challenges research scientists face in their careers. Because these rules are distilled by experts, they cover the most relevant professional challenges.

To formalize our collaborative proposal, we used Sandve and colleagues (2013) with *Ten Simple Rules for Reproducible Computational Research* [1], Michener (2015) with *Ten Simple Rules for Creating a Good Data Management Plan* [2], and Schwab and colleagues (2022) with *Ten Simple Rules for Good Research Practice* [3], which vigorously promote reproducible science. The simple rules provided by these articles efficiently helped us to successfully master the challenge of submitting our proposal on time and laid a firm formal basis for our collaborative project. For our project focused on reproducible science to actually be reproducible, these rules also proved essential.

In the process of proposal writing, we grouped the simple rules listed in [1–3] by the role they play in a cross-disciplinary project with two research partners from two distinct disciplines. As the proposal evolved, we referred to these rules several times until we arrived at the final version of our proposal, an approach that greatly impacted the design of the whole project. In fact, following these rules has efficiently transformed our initial intentions into the project outlined in Fig 1, which holds both partners equally responsible for reproducibility within their subprojects and for high-quality output, either data or software, that will be shared with the other partner. These rules also guided our in-depth discussions and helped us to clarify funder expectations and key points to highlight in the proposal narrative and in the Data Management Plan, thus greatly accelerating the decision-making process. Finally, the in-depth discussions we had about these rules promoted mutual understanding, trust, and respect between the teams involved in our cross-disciplinary project.

## Project overview

Fig 1 demonstrates our collaborative project with two equal partners, one from statistics and one from dental materials research, an applied science. The main goal of our cross-disciplinary collaboration is to develop and share standardized reproducible workflows for study design, calibrations, quality assurance and quality control, data collection, reproducible data analyses, dynamic reporting, and the manufacturing process of dental materials. To develop novel dental materials, the dental materials research partner conducts three types of experiments: preinvestigations, test groups, and final in vitro examinations. At each stage, in vitro data are generated, collected, and passed on to the statistical partner for analysis. To guarantee the reproducibility of the whole collaborative project, both partners must guarantee the reproducibility of their own subprojects.

The statistical partner uses both classical and Bayesian statistical methods and dynamic reporting tools in R (https://www.r-project.org/) to develop study designs, transparent sample size computation, data visualization, and reproducible statistical analyses. The partners communicate both directly and through data and reports. Our project uses GitHub (https://github.com/) to assure version control and Zenodo (https://zenodo.org/) to assure time stamping of datasets, software, and reports.

## Simple rules in practice

To design our project, we used all rules listed in [1–3]. As shown in Table 1, we assigned these rules to four sets of rules that pertain to general rules for the whole project (G), rules for dental materials research (D), rules for statistics (S), and rules for data and reports (R). The number at the end of each rule refers to the original article. Cursive indicates that we have tailored the original formulation to better fit our setting. Some of the rules appear twice in Table 1 because they apply to both the dental materials research partner and the statistical partner, but with a slightly different meaning. While we comment on the rules from the point of view of our project, more detailed explanations can be retrieved from the original articles [1–3]. In what follows, we demonstrate how we applied these rules to our cross-disciplinary collaborative project and substantiate why these rules are imperative for a scientific project to be reproducible.

**Table 1:** Rules from [1–3] used to design the project outlined in Fig 1.

G: General rules for the whole project	R: Rules for data and reports
G1: Determine the research sponsor requirements [2]	R1: Write a data management plan [3]
G2: Assign roles and responsibilities [2]	R2: Present a sound data storage and preservation strategy [2]
G3: Prepare a realistic budget [2]	R3: Describe how the data will be disseminated [2]
	R4: Make your research open [3]
**D: Rules for dental materials research**	**S: Rules for statistics**
D1: Specify your research question [3]	S1: For analyses that include randomness, note underlying random seeds [1]
D2: Identify the data to be collected [2]	S2: Always store raw data behind plots [1]
D3: Define how the data will be organized [2]	S3: Generate hierarchical analysis output, allowing layers of increasing detail to be inspected [1]
D4: Explain how the data will be documented [2]	S4: Record all intermediate results, when possible in standardized formats [1]
D5: Describe how data quality will be assured [2]	S5: Avoid manual data manipulation steps [1]
D6: Define the project’s data polices [2]	S6: *Follow good-practice research procedures* [3]
D7: Write and register a study protocol [3]	S7: Report all findings [3]
D8: Justify your sample size *(and the study design)* [3]	S8: Follow reporting guidelines [3]
D9: Reduce bias [3]	S9: For every result, keep track of how it was produced [1]
D10: Avoid questionable research practices [3]	S10: Connect textual statements to underlying results [1]
D11: Record all intermediate results, when possible in standardized formats [1]	S11: Be cautious with interpretations of statistical significance [3]
D12: For every result, keep track of how it was produced [1]	S12: Describe how data quality will be assured *(data = software, reports)* [2]
D13: Version control all *documents* [1]	S13: Define the project’s data polices *(data = software, reports)* [2]
D14: Avoid manual data manipulation steps [1]	S14: Archive the exact versions of all external programs used [1]
D15: Provide public access to scripts, runs, and results *(and data)* [1]	S15: Version control all custom scripts [1]
D16: Follow reporting guidelines [3]	S16: Provide public access to scripts, runs, and results [1]

Rules G1–G3 of Table 1 substantially impacted the proposal writing process. Following G1, we determined that our leading research sponsor would focus on the principles of Findable, Accessible, Interoperable, and Reusable (FAIR) data and require a sound data management plan upon submission, which may evolve throughout the project. To properly formulate the data management plan, we thoroughly scrutinized FAIR data principles. At this stage, we noticed strong synergy effects between the simple rules listed in [2] and the FAIR data principles. As G2 stipulates, we specified roles and responsibilities in the fields covered by each partner separately and for the communication between both partners. In accordance with rule G3, we allocated a generous budget to reproducible science to support the necessary efforts, time, and a well-qualified staff [2].

Following rules D1–D6, the dental materials research partner is responsible for the reproducibility of in vitro experiments conducted in three stages of preinvestigations, test groups, and final in vitro examinations. Each stage requires specifying a new research question (D1). Moreover, the types, sources, volumes, and coding formats of datasets collected at each stage must be specified (D2). In addition, the dental materials research partner must specify conventions for file naming, time stamping (D3), meta-data documentation strategy (D4), training, instrument calibration, verification tests for quality assurance and quality control (D5), and whether datasets may be publicly shared (D6).

Rules D7–D12 focus on additional responsibilities of the dental materials research partner. At each stage of the preinvestigations, test groups, and final in vitro examinations, the dental research partner must write a study protocol that clarifies the research question, the hypotheses, the study design, and the sample size, as well as provide a detailed plan of the statistical analyses (D7–D8). Moreover, the dental research partner must reduce bias by implementing standardized operating procedures, randomization, blinding, and good-practice research procedures (D9–D10). To guarantee reproducibility, the dental research partner must record all intermediate results in standardized formats (D11) and keep track of how every result was produced (D12). To achieve these goals, the dental materials research partner has to version control all documents (D13), avoid manual data manipulation steps (D14), provide public access to scripts, runs, results, and datasets (D15), and follow reporting guidelines (D16).

The rules S1–S11 of Table 1 remind the statistical partner to use random seeds (S1), always store data behind plots (S2), generate a hierarchical analysis output allowing layers of increasing detail to be inspected (S3), record all intermediate results such as reports and software in standardized formats (S4 = D11), avoid manual data manipulation within dynamic reports (S5), and follow good-practice research procedures (S6). The statistical partner is also reminded to report all statistical analyses (S7), follow reporting guidelines (S8 = D16), keep track of how results have been produced (S9 = D12), connect textual statements to underlying software, analysis, and results (S10), and cautiously interpret statistical significance (S11).

Further rules remind the statistical partner to specify how the quality of the software and reports will be assured (S12 = D5), define policies for licensing the software and reports (S13 = D6), archive the exact versions of all external programs used (S14), version control all scripts (S15 = D13), and provide public access to the software and reports generated by the project (S16 = D15). Although identical wording of several rules is used for both the statistical and the dental materials research partners, these rules apply to measurements and meta-data on the dental materials research side and to software, software documentation, and dynamic reports on the statistical side. Moreover, the rule S6 is a positive formulation of the original rule D10 of [3].

The communication between both equal partners in our project is supported by rules R1–R4, which apply to data comprising measurements, meta-data, software, software documentation, and dynamic reports. To efficiently handle these data, we must implement and constantly improve our data management plan (R1). Moreover, we must specify a sound data storage and preservation strategy, which includes backups and decisions about how long these data should be stored, protected, preserved, and kept usable for the future (R2). Finally, we also must define how these data will be disseminated (R3) with the primary focus on making our research open (R4).

## Example

As an example of how we established reproducibility specified in our proposal, consider one specific issue of sharing raw in vitro data between both partners, as represented by two upper green arrows in Fig 1. In a typical dental material project, Excel files would contain the data and these files would be saved on a local machine, complicating the process of tracking changes and making the information inaccessible to others. In our discussions of this problem, we referred to the rules listed in Table 1 several times, ultimately deciding that the Comma Separated Values [CSV] format would be a better default format for sharing in vitro data within the project. This CSV format would greatly facilitate immediate graphical visualization, different verification tests for QA/QC, and analyses developed by statisticians at each stage of preinvestigations, test groups, and final in vitro examinations. Raw in vitro data would be saved and regularly backed up on a server hosted by the dental research partner. Statisticians would be granted rights to access these raw in vitro data directly and they would use a version control framework to develop and share software and dynamic reports. We would share the in vitro data collected during the test group and the final in vitro examination stages through scientific publications that refer to open-access and open-source materials, providing data, software, and source code as supplementary material. Discussing and making such decisions early in the project saved time and streamlined the collaboration, helping us to avoid confusion in later stages. All remaining issues pertaining to the proposal and the Data Management Plan were similarly clarified following the rules listed in Table 1.

## Final thoughts

Practical rules from [1–3] listed in Table 1 focus on how to produce reproducible science that enhances the credibility of results and scientific integrity. These rules provided a firm basis for the design of our project outlined in Fig 1, but they could also provide guidance for other collaborative projects from a wide range of disciplines in formalizing research proposals and accurately specifying clear conditions for reproducible research within and between project partners. For example, D rules listed in Table 1 would also apply to any other experimental research project and S rules would also apply to any computational science or research software engineering project in almost any collaboration. In contrast, G and R rules govern the interactions between the two research partners here and, therefore, strongly depend on the specific characteristics of this cross-disciplinary project.

For the reproducible conduct of future projects in practice, we also believe that the list of rules in Table 1 could provide a clear scaffolding. Yet, we anticipate that this list is not exhaustive once a project reaches the implementation phase. Should our project be funded, we anticipate several important decisions that are not mentioned in Table 1 that both collaborators must make. These decisions are related to the (i) communication within the project; (ii) the open access to datasets, software, documentation, and dynamic reports; and (iii) the long-lasting, system-wide usability of the results of our project.

The modality of communication within our project will highly depend on whether the dental materials research team will directly use tools and dynamic reporting in R, and this decision has not yet been solidified. The open access to datasets, software, and results depends on the use of one free-of-charge, open-access, permanent repository for the version control and a quotable storage of datasets, reports, and software. Currently, the statistical partner uses GitHub (https://github.com/) to assure version control of the software and dynamic reports and Zenodo (https://zenodo.org/) for quotable time stamping of datasets, software, and dynamic reports. As indicated by one of the reviewers, OSF (https://osf.io) is a free-of-charge, open-access, permanent repository, which allocates persistent digital object identifiers and now also provides version control facilities, thus making this service highly relevant to our project. The long-lasting, system-wide usability of the results of our project depends on whether our software can be used on computers with different operating systems such as Windows, Mac, and Linux. We anticipate that these decisions and putting the rules listed in Table 1 into practice will require considerable efforts and time.

For further guidance with resolving such issues, it would be useful to have additional editorial articles from the “Ten Simple Rules” series. There are certainly experts who already have firsthand experience with collaborative projects that implement reproducible science and promote reusable and transparent research that is open for critical appraisal. Ideally, such experts will be willing to share their expertise in the form of simple rules for long-lasting, system-wide reproducible projects that efficiently communicate and provide safe and shareable datasets, software, and dynamic reports. We have attempted to illustrate here the strong impact that such simple rules from the “Ten Simple Rules” series have had on the design of our cross-disciplinary collaborative project focused on reproducible science. We believe that this systematic approach to design can also assist other cross-disciplinary collaborative projects from a wide range of disciplines and that such conversations can provide a common scaffolding for how to construct reliable and reproducible science.
